# A Structured Sparse Bayesian Channel Estimation Approach for Orthogonal Time—Frequency Space Modulation

**DOI:** 10.3390/e25050761

**Published:** 2023-05-06

**Authors:** Mi Zhang, Xiaochen Xia, Kui Xu, Xiaoqin Yang, Wei Xie, Yunkun Li, Yang Liu

**Affiliations:** 1 School of Communication Engineering, Army Engineering University of PLA, Nanjing 210007, China; 2 Unit 31105 of PLA, Nanjing 210042, China

**Keywords:** orthogonal time–frequency space modulation, integrated sensing and communication, channel estimation, structured sparse Bayesian learning

## Abstract

Orthogonal time–frequency space (OTFS) modulation has been advocated as a promising waveform for achieving integrated sensing and communication (ISAC) due to its superiority in high-mobility adaptability and spectral efficiency. In OTFS modulation-based ISAC systems, accurate channel acquisition is critical for both communication reception and sensing parameter estimation. However, the existence of the fractional Doppler frequency shift spreads the effective channels of the OTFS signal significantly, making efficient channel acquisition very challenging. In this paper, we first derive the sparse structure of the channel in the delay Doppler (DD) domain according to the input and output relationship of OTFS signals. On this basis, a new structured Bayesian learning approach is proposed for accurate channel estimation, which includes a novel structured prior model for the delay-Doppler channel and a successive majorization–minimization (SMM) algorithm for efficient posterior channel estimate computation. Simulation results show that the proposed approach significantly outperforms the reference schemes, especially in the low signal-to-noise ratio (SNR) region.

## 1. Introduction

Future mobile communication systems must be better suited to serve a variety of developing applications in environments with high levels of mobility, including low-orbit satellites, manned and unmanned aircraft, manned and unmanned vehicles, and high-speed trains [1,2,3]. In these high-mobility scenarios, mobile channels exhibit the characteristics of doubly dispersive channels due to the influence of the Doppler effect and the multipath propagation effect. Orthogonal frequency division multiplexing (OFDM) technology, which is currently widely used in fourth-generation mobile communication systems, fifth-generation mobile communication systems [4,5], wireless local area networks (WLANs), digital video broadcasting (DVB), and other broadband transmission systems, can overcome intersymbol interference (ISI) caused by time dispersion [6]. Of course, the studies on OFDM systems over doubly dispersive channel are also plentiful. Some researchers [7,8] have studied the channel estimation and data detection problems of OFDM systems over doubly dispersive channels, and the simulation results showed that the performance of the proposed method is close to the ideal case with perfect channel state information. However, due to the strict orthogonality among the subcarriers of OFDM systems, the orthogonality will be greatly destroyed by the channel frequency dispersion effect caused by frequency bias and fast time-varying channels. This results in serious intercarrier interference (ICI), which greatly degrades system performance and makes it impossible to provide efficient and reliable services. Therefore, OFDM is highly sensitive to frequency offset and channel frequency dispersion [9].

Orthogonal time–frequency space (OTFS) is a new modulation technology that can adapt to the time–frequency double dispersion propagation characteristics of channels in a high-speed mobile environment; it has attracted extensive attention in industry [10,11,12]. A time-varying channel in the time–frequency (TF) domain is changed into a time-invariant channel in the DD domain using OTFS modulation. After such a two-dimensional transformation, the transmitted data symbols are subjected to minor subcarrier interference, and all symbols in the same OTFS data frame experience slow fading independent of time selectivity. OTFS modulation is carried out in the DD domain.

At present, OTFS modulation has made certain research progress in many aspects, such as system performance analysis and modulation and demodulation algorithms. It is assumed in [10] that the ideal pulse-forming waveform satisfies orthogonal criteria in both time and frequency, but in reality, this is not possible because of Heisenberg’s uncertainty principle. As a result, the biorthogonality assumption was loosened, and an OFDM-based OTFS modulation system was presented in [13,14]. This system takes into account the cyclic prefix (CP) of each OFDM symbol in the OTFS frame as well as the non-ideal rectangular pulse-shaping waveform. This benefit stems from the simplicity of implementation, but it also introduces the issue of high out-of-band radiation causing interference with adjacent channels. At the moment, frequency local pulse shaping [15] and time-domain windowing technology [16] are the two major techniques for maximizing out-of-band attenuation by lowering out-of-band radiation. Compared with the TF domain, the advantage of the DD domain is that only a small area, which is based on the channel response that will be introduced in the second part of the system model, is needed to describe the channel, and it has stronger sparsity for better channel estimation.

Currently, the main technical research on OTFS modulation involves the estimation of channel state information and data detection after estimation. Ref. [17] proposed an embedded pilot-assisted channel estimation scheme. The delay-Doppler plane’s arrangement rules for data symbols, pilot symbols, and guard symbols were created to successfully prevent pilot symbols and data symbols from interfering with one another during each OTFS frame. The pilots were created for multipath channels with integer and fractional Doppler shifts using the OTFS modulation technology. However, if only one pilot symbol was inserted, it caused high peak-to-average-power ratio (PAPR) problems. For example, in this article, pilot power is greater than data power. In [18], a channel estimation and data detection framework based on a superposition pilot was proposed. This framework superimposes a low-power pilot on data symbols in the DD domain, thus increasing the space of data transmission and effectively improving the spectral efficiency of OTFS modulation transmission. Such a design induces high computation complexity, and the interference between data and pilot symbol also reduces the estimated accuracy.

As emphasized in [19], integer approximation cannot adequately simulate the channel’s noninteger delay and Doppler shift. Therefore, using an orthogonal matching pursuit approach based on binary refinement estimation, which has a tolerable computing complexity and much lower estimated normalized mean square error, has been suggested. According to the sparseness of the channel in the delay-Doppler domain, ref. [20] suggested a new pilot mode and a channel estimate method based on sparse Bayesian learning (SBL). In this new pilot pattern, the guard interval was not set, and the data and pilot had the same energy. To update the parameters in the previous model using the expectation maximum (EM) algorithm, a sparse Bayesian learning framework was provided. Channel estimation was treated as a sparse recovery problem. To a certain extent, the pilot symbol power cost was reduced, as was the pilot symbol power consumption, and the noise interference was weakened.

In this paper, to reduce the pilot cost and improve the accuracy of channel estimation, we treat the channel problem of estimating the DD domain as a sparse recovery problem and derive the sparse model expression from the input–output relationship of the DD domain. We provide a new sparse Bayesian framework based on the channel’s sparse features, and the block successive majorization–minimization (SMM) technique is employed to handle the problem of the new prior structure. The simulation results show that, in comparison to existing reference approaches, our suggested method greatly reduces the noise interference in the situation of a low signal-to-noise ratio.

The rest of the paper is organized as follows. The OTFS modulation system model is described in Section 2. The structured sparse Bayesian approach for channel estimation is presented in Section 3. The simulation results are reported in Section 4, and Section 5 concludes the paper.

## 2. System Model

**Figure 1:**
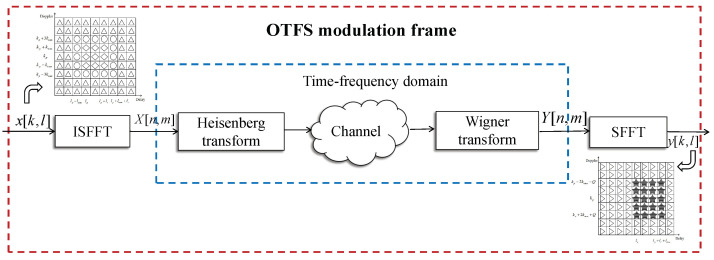
OTFS modulation frame.

We consider an OTFS modulation system with *M* symbols and *N* subcarriers. The transmitter and receiver are equipped with a single antenna. The OTFS modulation frame is shown in Figure 1.

### 2.1. OTFS Modulation and Demodulation

The transmitter transforms the information symbol $x\left[k,l\right]\in C^{M\times N}$ from the DD domain to the time–frequency domain using the inverse symplectic finite Fourier transform (ISFFT).

(1) $$X\left[n,m\right]=\frac{1}{\sqrt{MN}}\sum_{k=0}^{N}\sum_{l=0}^{M}x\left[k,l\right]e^{j2\pi (\frac{nk}{N}-\frac{ml}{M})}$$

At this time, X[n,m] generates a time waveform s(t) using a transmission pulse $g_{tx}\left(t\right)$. This transform is called the Heisenberg transform.

(2) $$s\left(t\right)=\sum_{n=0}^{N}\sum_{m=0}^{M}X\left[n,m\right]g_{tx}\left(t\right)e^{j2\pi m\Delta f(t-nT)}$$

where Δf and *T* are the subcarrier spacing and symbol period, respectively.

After that, the signal s(t) is transmitted through a fast time-varying channel with a complex baseband channel impulse response h(τ,ν), which specifies the channel response to an impulse with delay τ and Doppler ν.

(3) $$r\left(t\right)=\int \int h\left(\tau ,\nu \right)s\left(t-\tau \right)e^{j2\pi \nu \left(t-\tau \right)}$$

The channel only has a few parameters in the delay-Doppler domain. The sparse representation of channel h(τ,ν) is given as

(4) $$h\left(\tau ,\nu \right)=\sum_{i=1}^{P}h_{i}\delta \left(\tau -\tau_{i}\right)\delta \left(\nu -\nu_{i}\right)$$

where *P* denotes the number of paths for propagation. δ() denotes the Dirac delta function, whereas $h_{i}$, $\tau_{i}$ and $\nu_{i}$ stand for the path gain, delay, and Doppler shift associated with the *i*-th path, respectively. We choose the delay and Doppler taps for the *i*-th path as follows:

(5) $$\tau_{i}=\frac{l_{\tau_{i}}}{M\Delta f},\nu_{i}=\frac{k_{\nu_{i}}+\kappa_{\nu_{i}}}{NT}$$

where the integers $l_{\tau_{i}}$, $k_{\nu_{i}}$ represent the delay tap and Doppler tap indices corresponding to the delay $\tau_{i}$ and Doppler frequency $\nu_{i}$, respectively, and $\kappa_{\nu_{i}}\in \left[-0.5,0.5\right]$. We do not need to examine the impact of the fractional delay because the resolution of the time axis is sufficient to approximate the path delay to the nearest integer grid point.

The received signal r(t) is given by the Wigner transform and is realized by the receiving matching filter of impulse response $g_{rx}$, obtaining the time–frequency domain symbol Y[n,m].

(6) $$Y\left[n,m\right]=\int r(t)g_{rx}\left(t-nT\right)e^{j2\pi m\Delta f(t-nT)}dt$$

Finally, the symplectic finite Fourier transform (SFFT) is used to convert the information symbols Y[n,m] in the TF domain into the symbols y[k,l] in the DD domain.

When the transmitter pulse $g_{tx}$ and the receiver pulse $g_{rx}$ are ideal pulse functions, the relationship between the transmitted signal and the received signal in the delay-Doppler domain can be written as

(7) $$\begin{array}{cc} y\left[k,l\right] & =\sum_{i=1}^{P}\sum_{k^{{}^{\prime }}={\left[k-k_{\nu_{i}}-Q\right]}_{N}}^{{\left[k-k_{\nu_{i}}+Q\right]}_{N}}\\ \; & \left(\frac{e^{-j2\pi \left(k-k_{\nu_{i}}-k^{{}^{\prime }}-\kappa_{\nu_{i}}\right)}-1}{Ne^{-j\frac{2\pi }{N}\left(k-k_{\nu_{i}}-k^{{}^{\prime }}-\kappa_{\nu_{i}}\right)}-N}\right)+v\left[k,l\right]\\ \; & =\sum_{i=1}^{P}\sum_{q=-Q}^{Q}\left(\frac{e^{-j2\pi \left(-q-\kappa_{\nu_{i}}\right)}-1}{Ne^{-j\frac{2\pi }{N}\left(-q-\kappa_{\nu_{i}}\right)}-N}\right)h_{i}e^{-j2\pi \nu_{i}\tau_{i}}\\ \; & \times x\left[{\left[k-k_{\nu_{i}}+q\right]}_{N},{\left[l-l_{\tau_{i}}\right]}_{M}\right]+v\left[k,l\right] \end{array}$$

where v[k,l] denotes the system noise in the DD domain. We set the proper *Q* to counteract the impact of fractional Doppler. The delay-Doppler plane is discretized to an M×N grid. According to Equation (7), due to the influence of the doubly dispersive channel, the signal received at the *k*-th Doppler point is subjected to the superposition of signals from the 2Q+1 (k−Q to k+Q) Doppler points. In other words, the received signal y[k,l] is a linear combination of the sent signal $\Omega =\sum_{i=1}^{P}2Q+1$.

### 2.2. Sparse Delay-Doppler Domain Channel Model

To implement channel estimation, we place $L=\left(2k_{max}+2Q+1\right)\left(l_{max}+1\right)$ pilot symbols $x_{p}\left[k,l\right]$ in the DD domain. The greatest delay $\tau_{max}$ and Doppler $\nu_{max}$ related to the delay and Doppler taps are defined as $k_{max}$ and $l_{max}$. In addition, we formulate Equation (7) in a different form, as follows:

(8) $$y\left[k,l\right]=\sum_{k^{\prime }=-k_{max}-Q}^{k_{max}+Q}\sum_{l^{\prime }=0}^{l_{\max}}h\left[k^{\prime },l^{\prime }\right]x\left[k-k^{\prime },l-l^{\prime }\right]$$

After a series of formula calculations, we finally obtain the matrix form of the formula as

(9) y=Φh+V

where

(10) $$h=\left[\begin{array}{c} h[0]\\ \vdots \\ h[l_{max}] \end{array}\right]$$

(10a) $$h\left[l^{\prime }\right]=\left[\begin{array}{c} h\left[-k_{max}-Q,l^{\prime }\right]\\ \vdots \\ h\left[k_{max}+Q,l^{\prime }\right] \end{array}\right]$$

where $l^{{}^{\prime }}\in \left[0,l_{max}\right]$

(11) $$\begin{array}{cc} \Phi & =\sum_{l^{{}^{\prime }}=0}^{l_{max}}x_{k,l-l^{{}^{\prime }}}^{T}\\ \; & =\sum_{l^{{}^{\prime }}=0}^{l_{max}}\left[\begin{array}{c} x[k-\left(-k_{max}-Q\right),l-l^{{}^{\prime }}]\\ \vdots \\ x[k-\left(k_{max}+Q\right),l-l^{{}^{\prime }}] \end{array}\right]\\ & =\left[\begin{array}{c} X_{k^{{}^{\prime }},l-0}^{T}\\ \vdots \\ X_{k^{{}^{\prime }},l-l_{max}}^{T} \end{array}\right]=\left[\begin{array}{c} X_{k^{{}^{\prime }},l^{{}^{\prime }}}^{T}\\ \vdots \\ X_{k^{{}^{\prime }},l^{{}^{\prime }}}^{T} \end{array}\right] \end{array}$$

where $\Phi \in C^{S\times L}$, $h\in C^{L\times 1}$, and $V\in C^{S\times 1}$ are the received signal, transmitted signal. and Gaussian noise in the DD domain, respectively. *S* is the size of the channel estimation area. A simple proof is provided in Appendix A.

## 3. Structured Sparse Bayesian Approach for Channel Estimation

In this section, according to the sparse characteristics of the channel in the DD domain, we use Equation (8) to introduce the improved sparse Bayesian algorithm in detail. First, we briefly introduce the design of the pilot pattern at the receiver and transmitter. Second, we consider the sparse characteristics of the channel and design an improved sparse Bayesian algorithm. Finally, the complexity of the proposed algorithm is discussed.

### 3.1. Pilot Placement and Pattern Design

The power of the pilot symbol in the embedded pilot channel estimate approach is frequently substantially larger than that of the data symbol, which is unrealizable in actual applications. Only inserting one pilot symbol will result in a high PARP issue [17]. Meanwhile, unlike [20], we have set up a protection interval symbol to avoid interference between data and pilot symbols. Here, we set the pilot symbol power to be the same as the data symbol power, transforming the channel estimation problem into a sparse channel recovery problem. This not only effectively solves the problem of high pilots power but also avoids interference between data that affects estimation results.

**Figure 2:**
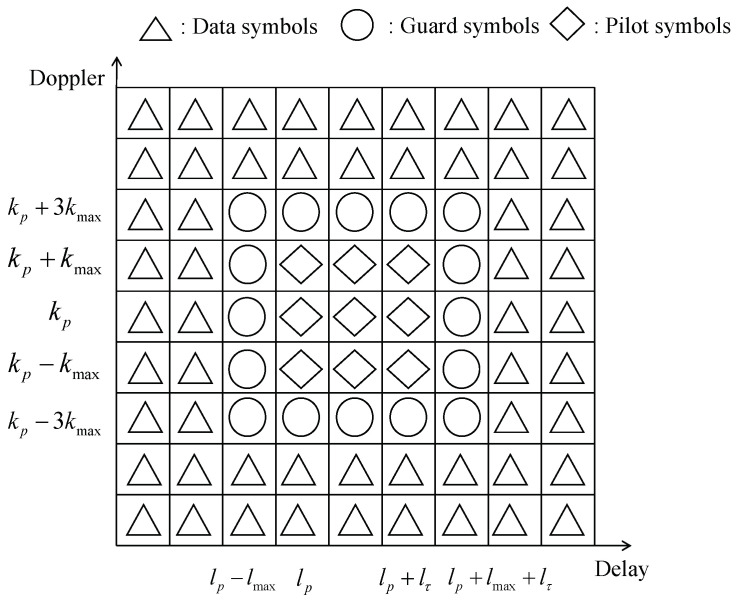
Tx symbols pattern.

We arrange the pilot and data symbols in the delay-Doppler grid for OTFS frame transmission, as shown in Figure 2.

We select $k\in [k_{p}-k_{max},k_{p}-k_{max}]$ and $l\in [l_{p},l_{p}+l_{\tau }]$ as the pilot symbols. The other fields are guard symbols and data symbols.

(12) $$x\left[k,l\right]=\left\{\begin{array}{cc} x_{p}\left[k,l\right] & \begin{array}{c} k_{p}-k_{\max}\leq k\leq k_{p}-k_{\max}\\ l_{p}\leq l\leq l_{p}+l_{\tau } \end{array}\\ 0 & \begin{array}{c} k_{p}-3k_{\max}\leq k\leq k_{p}-3k_{\max}\\ l_{p}-l_{\max}\leq l\leq l_{p}+l_{\tau }+l_{\max} \end{array}\\ x_{d}\left[k,l\right] & otherwise \end{array}\right.$$

**Figure 3:**
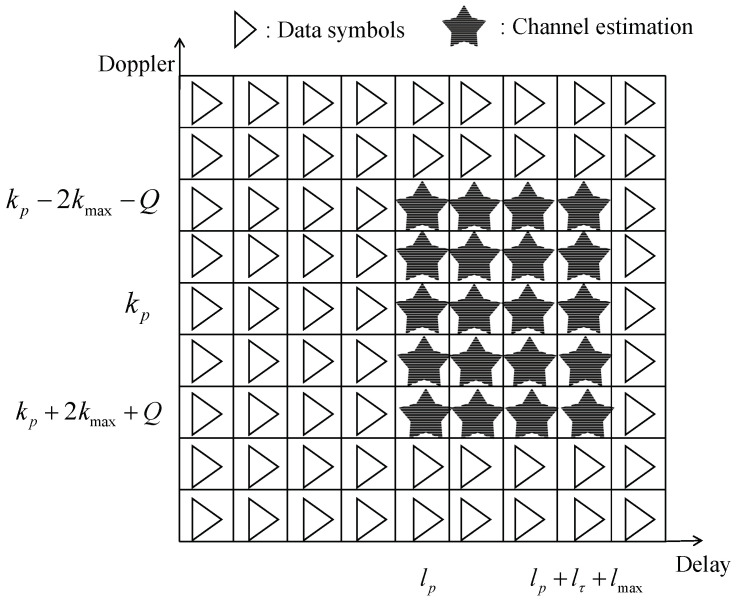
Rx symbols pattern.

Usually, $\left(k_{p},l_{p}\right)$ are placed onto $\left(\frac{N}{2},\frac{M}{2}\right)$. $l_{\tau }$ is used to control the proportion of pilots in the whole transmission symbol. The final channel estimation region is set to $k\in [k_{p}-k_{max}-Q,k_{p}-k_{max}+Q]$ and $l\in [l_{p},l_{p}+2l_{\tau }]$. In other words, the size of the channel estimation section $S=\left(4k_{max}+2Q+1\right)\left(l_{max}+l_{\tau }+1\right)$, as shown in Figure 3.

### 3.2. Structured Sparse Bayesian Channel Estimation Problem Formulation

**Figure 4:**
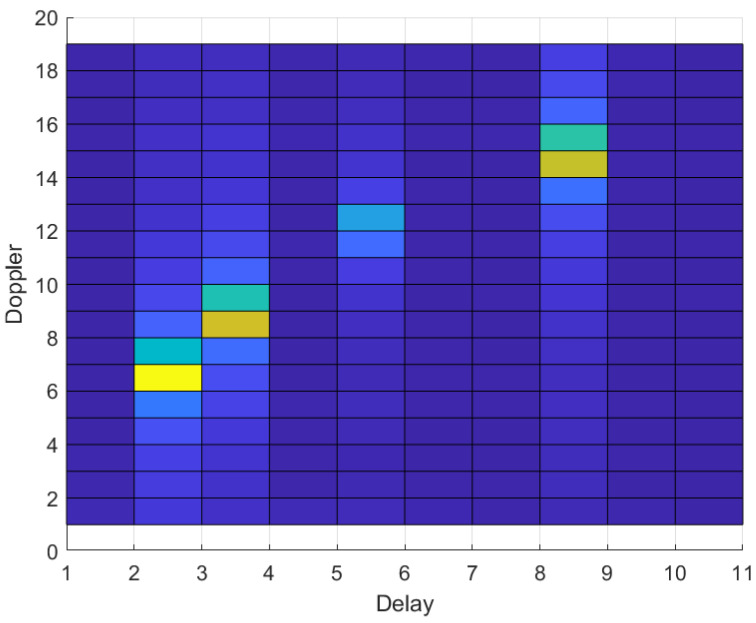
Characteristics of sparsity.

We present the channel coefficient characteristics in the DD domain in Figure 4. According to the characteristics of channel sparsity, we propose a new estimation algorithm.

We provide an example of the DD domain channel corresponding to (7). As shown in Figure 4, the DD domain channel only has 4 non-zero responses over the whole DD channel, whose coordinates align with the associated delay and Doppler shifts. Therefor, estimating channel h can be viewed as a sparse recovery problem. Although the traditional sparse Bayesian algorithm can be used here, there are only values on each path according to the channel sparsity in the DD domain, and when a value is detected, it means that there are values before and after the value, so the traditional algorithm does not reflect this feature.

According to the sparse characteristics of the channel, we propose an improved sparse Bayesian algorithm, as follows:

(13) $$\begin{array}{cc} p(h|\alpha_{l,k},\gamma ) & =Nc(h|0,diag\left(\gamma \right)\times diag\left(D_{l}\right)) \end{array}$$

where $\gamma =\{\gamma_{1},\gamma_{2},\ldots ,\gamma_{l_{\max}+1}\}$, $D_{l}$ is a diagonal matrix whose n-th diagonal element is given by

(14) $${\left[D_{l}\right]}_{k,k}=\left\{\begin{array}{c} \alpha_{l,1}+\alpha_{l,2}\\ \alpha_{l,k-1}+\alpha_{l,k}+\alpha_{l,k+1}\\ \alpha_{l,k-1}+\alpha_{l,k+1} \end{array}\right.\begin{array}{c} k=1\\ 2\leq k\leq 2k_{\max}+2Q\\ k=2k_{\max}+2Q+1 \end{array}$$

where $\alpha_{l}=\left\{\alpha_{l,1},\alpha_{l,2},\ldots ,\alpha_{l,2k_{\max}+2Q+1}\right\}$, $D_{l}=\left\{D_{1},D_{2},\ldots ,D_{{}_{l_{\max}+1}}\right\}]$.

As above, in the design of the diagonal matrix $D_{l}$, the design of the parameter $\alpha_{l,k}$ not only affects the *k*th element of channel h to be estimated; it also affects its neighbouring elements. As $\alpha_{l,k}$ tends to infinity, the *k*-th, (k+1)th, and (k−1)th elements are simultaneously driven to zero. The distribution of zero elements exhibits a blocky distribution. On the other hand, by dividing the whole h into $l_{max}$ column vectors, the function of γ is used to distinguish the difference between the different column vectors.

According to Equation (9), the posterior distribution of h can be expressed as

(15) $$p\left(h|y,\alpha_{l,k},\gamma \right)\propto p\left(y|h\right)p\left(h|\alpha_{l,k},\gamma \right)$$

where the likelihood function of *y* is p(y|h), which is

(16) $$p\left(y|h\right)=Nc\left(y|\Phi h,\sigma^{2}I\right)$$

By substituting Equations (13) and (16) into Formula (15), it is not difficult to prove that this is a posterior distribution that follows a complex Gaussian distribution through mathematical calculation, and the mean and covariance are as follows:

(17) $$\begin{array}{cc} \; & \mu =\frac{1}{\sigma^{2}}\sum \Phi^{H}y\\ \; & \sum ={\left(diag\left\{\gamma \right\}\times diag\left\{D_{l}\right\}+\frac{1}{\sigma^{2}}\Phi^{H}\Phi \right)}^{-1} \end{array}$$

Here, as in the traditional sparse Bayesian algorithm, we express the Gamma hyperprior with respect to α as follows:

(18) $$\begin{array}{cc} p(\alpha ) & =\prod_{k=1}^{2k_{\max}+2Q+1}Gam\left(\alpha_{l,k}|a,b\right)\\ \; & =\prod_{k=1}^{2k_{\max}+2Q+1}\frac{b^{a}}{\Gamma (a)}{\alpha_{l,k}}^{a}\exp\left(-b\alpha_{l,k}\right) \end{array}$$

where *a* and *b* are fixed parameters. In this section, we introduce the small positive numbers a and b $\left(a=b=10^{-4}\right)$. Such a configuration encourages a flat prior for α.

We also select a Dirichlet hyperprior for γ,

(19) $$p\left(\gamma \right)=C\left(u\right)\prod_{l=1}^{l_{\max}+1}\gamma^{u_{l}}$$

where $u=[u_{1},u_{2},\ldots ,u_{l_{max}}]$ is a fixed parameter. $C\left(u\right)=\frac{\Gamma (\sum_{l=1}^{l_{\max}}u_{l})}{\Gamma \left(u_{1}\right)\cdots \Gamma \left(u_{l}\right)}$ is the normalization constant. The role of γ is to model the relative difference between different sub-vectors u. By exploiting the common sparsity structure, the suboptimal solutions in the conventional methods that predict a value of zero for one element of $h_{\left[l\right]}$ and predict a nonzero value for the element of $h_{\left[l^{{}^{\prime }}\neq l\right]}$ in the same position can be eliminated. Since the expectation of γ with respect to the distribution (19) is given by $E[\gamma ]=u_{l}/\sum_{l^{{}^{\prime }}=1}^{l_{\max}}u_{l^{{}^{\prime }}}$, we can interpret the parameter u, which provides a initial guess as to the relative difference between the sub-modules of h. The parameter that provides an initial estimation of the relative difference between the submodules of h can be interpreted as u. Note that a default setup for u can be $u_{l}=1$ for $l=1,\ldots ,l_{max}$, which indicates an initial assumption of different delay modules.

The maximum posterior (MAP) estimation of h can be derived by its posterior mean in Equation (17) as long as $\alpha_{l,k}$. As a result, in the sections that follow, we concentrate on obtaining the ideal $\alpha_{l,k}$ and γ by solving the MAP issue.

(20) $$\begin{array}{cc} \left\{\alpha_{l,k}^{opt},\gamma^{opt}\right\} & =\arg\max_{\alpha_{l,k},\gamma }p\left(\alpha_{l,k},\gamma |y\right)\\ \; & =\arg\min_{\alpha_{l,k},\gamma }-\log p\left(\alpha_{l,k},\gamma ,y\right) \end{array}$$

Since we proposed a new structured prior model for h, it is clear that the above problems cannot be solved if we continue to use the traditional sparse Bayesian algorithm. Therefore, we provide a solution using the block SMM algorithm.

#### SMM Algorithm for Posterior Channel Estimate Evaluation

The proposed block SMM algorithm is similar to the expected EM algorithm. It has two main steps. The first step is to replace the optimization step with the expected step, and its purpose is to find the alternative function of the objective function, that is, the local approximate solution. The second step is the minimization step, in which the alternative function found in the first step is minimized. While the block SMM technique divides the parameters to be estimated into many blocks for alternate optimization, the EM algorithm updates all parameters concurrently. This is helpful when solving structured SBL is the only obstacle to successfully minimizing all of the proxy function’s parameters.

At this point, we assume that $G(\alpha_{l,k},\gamma ,y|{\hat{\alpha }}_{l,k},\hat{\gamma })$ is the alternative function in Step 1, where $\left({\hat{\alpha }}_{l,k},\hat{\gamma }\right)$ is the estimation of $\left(\alpha_{l,k},\gamma \right)$. If $G(\alpha_{l,k},\gamma ,y|{\hat{\alpha }}_{l,k},\hat{\gamma })$ satisfies the following condition:

(21) $$\begin{array}{c} \begin{array}{c} G\left(\alpha_{l,k},\gamma ,y|{\hat{\alpha }}_{k},\hat{\gamma }\right)\geq -\log p\left(\alpha_{l,k},\gamma ,y\right),\forall \alpha_{l,k},\gamma \\ G\left({\hat{\alpha }}_{l,k},\hat{\gamma },y|{\hat{\alpha }}_{l,k},\hat{\gamma }\right)=-\log p\left({\hat{\alpha }}_{l,k},\hat{\gamma },y\right)\\ {\left.\frac{\partial G(\alpha_{l,k},\hat{\gamma },y|{\hat{\alpha }}_{l,k},\hat{\gamma })}{\partial \alpha_{l,k}}\right|}_{\alpha_{l,k}={\hat{\alpha }}_{l,k}}={\left.\frac{\partial \{-\log p\left(\alpha_{l,k},\hat{\gamma },y\right)\}}{\partial \alpha_{l,k}}\right|}_{\alpha_{l,k}={\hat{\alpha }}_{l,k}}\\ {\left.\frac{\partial G({\hat{\alpha }}_{l,k},\gamma ,y|{\hat{\alpha }}_{l,k},\hat{\gamma })}{\partial \gamma }\right|}_{\gamma =\hat{\gamma }}={\left.\frac{\partial \{-\log p\left({\hat{\alpha }}_{l,k},\gamma ,y\right)\}}{\partial \gamma }\right|}_{\gamma =\hat{\gamma }} \end{array} \end{array}$$

In addition, $\left\{\alpha_{l,k},\gamma \right\}$ follows the following rules in each iteration:

(22) $$\begin{array}{c} {\hat{\alpha }}_{l,k}^{\left(i+1\right)}=\arg\max_{\alpha_{l,k}}G\left(\alpha_{l,k},{\hat{\gamma }}^{\left(i\right)},y|{\hat{\alpha }}_{l,k}^{\left(i\right)},{\hat{\gamma }}^{\left(i\right)}\right)\\ {\hat{\gamma }}^{\left(i+1\right)}=\arg\max_{\alpha_{l,k}}G\left({\hat{\alpha }}_{l,k}^{\left(i+1\right)},\gamma ,y|{\hat{\alpha }}_{l,k}^{\left(i\right)},{\hat{\gamma }}^{\left(i\right)}\right) \end{array}$$

where $\left\{{{\hat{\alpha }}_{l,k}}^{\left(i\right)},{\hat{\gamma }}^{\left(i\right)}\right\}$ represents $\alpha_{l,k},the\;\gamma$ estimated value in the *i*-th iteration.

After a series of mathematical calculations, the specific algorithm is as follows.

(1) Step 1: A substitute for the objective function $-\log p(\alpha_{l,k}.\gamma_{,}y)$ is required at the corresponding step of the (i+1)th iteration. In this study, we make use of the surrogate function proposed by [21], which meets the requirements stated in (21), that is, a simple proof is provided in Appendix B.

(23) $$\begin{array}{cc} \; & G(\alpha_{l,k},\gamma ,y|{\hat{\alpha }}_{l,k}^{\left(i\right)},{\hat{\gamma }}^{\left(i\right)})=\\ \; & -\int p(h|y,{\hat{\alpha }}_{l,k}^{\left(i\right)},{\hat{\gamma }}^{\left(i\right)})\log\frac{p(\alpha_{l,k},\gamma ,h,y)}{p(h|y,{\hat{\alpha }}_{l,k}^{\left(i\right)},{\hat{\gamma }}^{\left(i\right)})}dh\\ \; & \geq -\log p(\alpha_{l,k},\gamma ,y) \end{array}$$

where the final inequation obeys Jensen’s inequality, and when the inequation satisfies $\left(\alpha_{l,k},\gamma \right)=\left(\alpha_{l,k}^{\left(i\right)},{\hat{\gamma }}^{\left(i\right)}\right)$, the equal sign is true. By substituting (15) into (23), we can obtain

(24) $$\begin{array}{cc} G(\alpha_{l,k},\gamma ,y|{\hat{\alpha }}_{l,k}^{\left(i\right)},{\hat{\gamma }}^{\left(i\right)}) & =-\log p\left(\alpha_{l,k}\right)-\log p\left(\gamma \right)\\ \; & -E_{\left[\log p\left(h|\alpha_{l,k},\gamma \right)\right]}+const \end{array}$$

where the expectation is in relation to $p(h|y,{\hat{\alpha }}_{l,k}^{\left(i\right)},{\hat{\gamma }}^{\left(i\right)})$. const denotes that it is not related to $\alpha_{l,k}$ or γ.

(2) Step 2: To minimize the surrogate function, the parameters $\alpha_{l,k}$ are changed sequentially in the corresponding step of the (i+1)th iteration.

To update $\alpha_{l,k}$; γ is regarded as a constant when $\alpha_{l,k}$ is estimated. By substituting (13) and (18) into (24), we obtain

(25) $$\begin{array}{cc} \; & G(\alpha_{l,k},{\hat{\gamma }}^{\left(i\right)},y|{\hat{\alpha }}_{l,k}^{\left(i\right)},{\hat{\gamma }}^{\left(i\right)})\\ \; & =-\sum_{k=1}^{2k_{\max}+2Q+1}\left(a\log\alpha_{l,k}-b\alpha_{l,k}\right)\\ \; & -\log\left|diag\left(\gamma \right)\times diag\left(D_{l}\right)\right|\\ \; & +tr(\left(diag\left(\gamma \right)\times diag\left(D_{l}\right)\right)\times \left(\hat{\sum }+\hat{\mu }{\hat{\mu }}^{H}\right))\\ \; & +const \end{array}$$

where Equation (17) is used to compute $\hat{\Sigma }$ and $\hat{\mu }$, and $\left(\alpha_{l,k},\gamma \right)$ is the result obtained from the *i*-th iteration. Next, we consider the derivative of (25) with regard to $\alpha_{l,k}$ to analyse the optimality condition.

(26) $$\begin{array}{cc} \frac{\partial G(\alpha_{l,k},{\hat{\gamma }}^{\left(i\right)},y|{\hat{\alpha }}_{l,k}^{\left(i\right)},{\hat{\gamma }}^{\left(i\right)})}{\partial \alpha_{l,k}} & =\frac{a}{\alpha_{l,k}}-b-{\hat{\gamma }}^{\left(i\right)}g\\ \; & +(\xi_{k-1}+\xi_{k}+\xi_{k+1}) \end{array}$$

where

(27) $$\begin{array}{cc} g & =\sum_{j=1}^{L}\\ \; & \left\{\begin{array}{c} {\left[\tilde{\Sigma }\right]}_{\left[j,j\right]}+{\left|{\left[\tilde{\mu }\right]}_{j}\right|}^{2}\\ +{\left[\tilde{\Sigma }\right]}_{\left[{\left[j+MAX\right]}_{L},{\left[j+MAX\right]}_{L}\right]}+{\left|{\left[\tilde{\mu }\right]}_{{\left[j+MAX\right]}_{L}}\right|}^{2}\\ +{\left[\tilde{\Sigma }\right]}_{\left[{\left[l_{\max}\cdot MAX+j\right]}_{L},{\left[l_{\max}\cdot MAX+j\right]}_{L}\right]}\\ +{\left|{\left[\tilde{\mu }\right]}_{{\left[l_{\max}\cdot MAX+j\right]}_{L}}\right|}^{2} \end{array}\right\} \end{array}$$

(28) $$\xi_{k}=\frac{1}{\alpha_{l,k-1}+\alpha_{l,k}+\alpha_{l,k+1}}$$

Specifically, when k=1

(28a) $$\xi_{k}=\frac{1}{\alpha_{1}+\alpha_{2}}$$

when k= MAX

(28b) $$\xi_{k}=\frac{1}{\alpha_{MAX-1}+\alpha_{MAX}}$$

where $MAX=2k_{max}+2Q+1$.

Because $\alpha_{l,k}$ is coupled with the adjacent terms $\alpha_{l,k-1}$ and $\alpha_{l,k+1}$ in the term $\xi_{k}$, we are unable to acquire $\alpha_{l,k}$ by setting (26) to zero. Here, we have $\alpha_{l,k}$, $\alpha_{l,k+1}$, $\alpha_{l,k-1}$. The expression for Equation (26) is

(29) $$\frac{\partial G(\alpha_{l,k},{\hat{\gamma }}^{\left(i\right)},y|{\hat{\alpha }}_{l,k}^{\left(i\right)},{\hat{\gamma }}^{\left(i\right)})}{\partial \alpha_{l,k}}=\frac{a}{\alpha_{l,k}}-b+\frac{1}{\alpha_{l,k}}-{\hat{\gamma }}^{\left(i\right)}g$$

By setting (29) to zero, we can obtain

(30) $${{\hat{\alpha }}_{l,k}}^{\left(i+1\right)}=\frac{a+1}{{\hat{\gamma }}^{\left(i\right)}g+b}$$

Here, we do not address special cases (when k=1 and k= MAX).

To update γ, by leaving out the terms that are unrelated to γ and inserting (13) and (19) into (24), we obtain

(31) $$\begin{array}{cc} \; & G({\hat{\alpha }}_{l,k}^{\left(i+1\right)},\gamma ,y|{\hat{\alpha }}_{l,k}^{\left(i\right)},{\hat{\gamma }}^{\left(i\right)})=\\ \; & -\sum_{l=0}^{l_{\max}}u\log\gamma -\log\left|diag\left(\gamma \right)\times diag\left(D_{l}\right)\right|\\ \; & +tr(\left(diag\left(\gamma \right)\times diag\left(D_{l}\right)\right)\times \left(\hat{\sum }+\hat{\mu }{\hat{\mu }}^{H}\right))\\ \; & +const \end{array}$$

where $\alpha_{l,k}$ is fixed at its latest estimation $\alpha_{l,k}=\alpha_{l,k}^{\left(i+1\right)}$. In relation to γ, the derivative of (31) can be written as

(32) $$\begin{array}{cc} \; & \frac{\partial G({\hat{\alpha }}_{l,k}^{\left(i+1\right)},\gamma ,y|{\hat{\alpha }}_{l,k}^{\left(i\right)},{\hat{\gamma }}^{\left(i\right)})}{\partial \alpha_{l,k}}\\ \; & =\frac{l_{\max}+u}{\gamma }\\ \; & -\left({\hat{\alpha }}_{l,k}^{\left(i+1\right)}+{\hat{\alpha }}_{l,k-1}^{\left(i+1\right)}+{\hat{\alpha }}_{l,k+1}^{\left(i+1\right)}\right)\times \left(\sum_{j=1}^{L}{\left[\hat{\Sigma }\right]}_{\left[j,j\right]}+{\left|{\left[\hat{\mu }\right]}_{j}\right|}^{2}\right) \end{array}$$

The best value of γ can be attained by setting (32) to zero as

(33) $$\begin{array}{cc} {\hat{\gamma }}_{}^{\left(i+1\right)} & =\frac{l_{\max}+u}{{\hat{\alpha }}_{l,k}^{\left(i+1\right)}+{\hat{\alpha }}_{l,k-1}^{\left(i+1\right)}+{\hat{\alpha }}_{l,k+1}^{\left(i+1\right)}}\\ \; & \times {\left(\sum_{j=1}^{L}{\left[\hat{\Sigma }\right]}_{\left[j,j\right]}+{\left|{\left[\hat{\mu }\right]}_{j}\right|}^{2}\right)}^{-1} \end{array}$$

Step 1 and Step 2 are performed iteratively until convergence. Algorithm 1 is a summary of the SMM algorithm presented in this paper.
**Algorithm 1** SMM algorithm for structured SBL**Input:** threshold ε, maximum iterations $iter_{max}$    Setting the initial value $\alpha_{k}$ and γ as the first loop value.**Output:** Channel estimation h.1:Step 1: the *i*-th iteration $\alpha_{l,k}^{\left(i\right)},{\hat{\gamma }}^{\left(i\right)}$ as fixed parameters $\alpha_{l,k},\gamma$. Update the posterior mean and covariance matrix of h using (16).2:Step 2: Update $\alpha_{l,k}$ by (29).              Update γ by (32).3:Convergence of judgment:Let $h^{i+1}$ denote the estimation of h after the (i+1)th iteration.4:**if**${∥h^{i+1}-h^{i}∥}^{2}\leq \varepsilon$ **or** $i+1=iter_{max}$ **then**5:   Output $\hat{h}$6:**else**7:   Set i=i+1 and runtun Step 18:**end if**

Afterwards, the algorithm will be further studied and introduced into the MIMO system. The dimensions of the channel change from the SISO system to the MIMO system, and the channel dimension increases linearly with the increase of the number of antennas. The sparse structure of the channel will therefore expand from one-dimensional to multi-dimensional.

The calculation of the posterior μ and covariance matrix Σ of h in the MA-step in Algorithm 1 accounts for the majority of the computing effort for each iteration $iter_{max}$. Therefore, the maximum computational complexity order of the structured SBL-based channel estimation algorithm is $O\left(iter_{\max}L^{3}S^{3}\right)$.

## 4. Simulation Results

In this section, we verify the performance of our proposed algorithm by the normalized mean square error (NMSE). The NMSE was defined as ${∥h-\hat{h}∥}_{2}^{2}/{∥h∥}_{2}^{2}$. We set the parameters as follows (see Table 1): the number of symbols N=64, the number of subcarriers M=128, the carrier frequency was 15 kHz, and the subcarrier frequency was $4\times 10^{9}$ Hz. The maximum Doppler shift varied according to the speed, and the maximum delay value was $l_{\tau }=10$. $k_{max}$ was a parameter related to speed.

**Figure 5:**
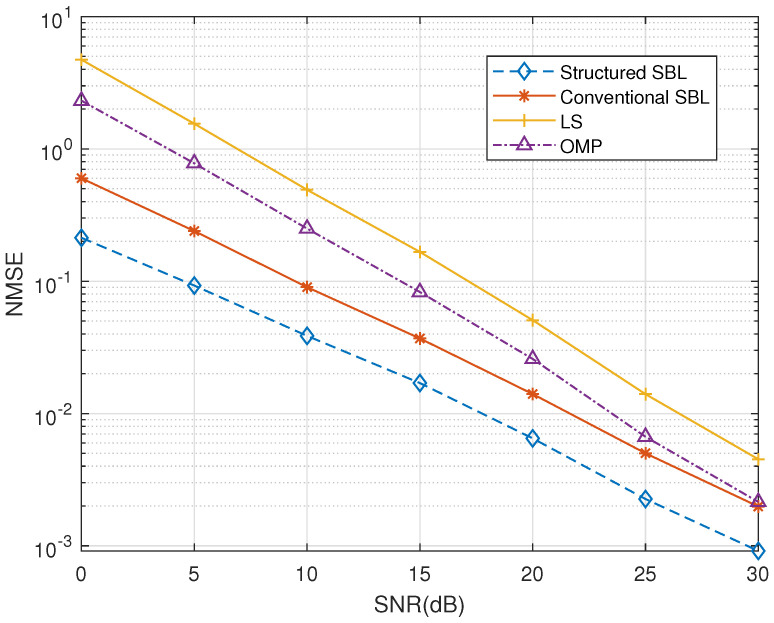
Normalized MSE versus SNR.

First, we compared the performance of different algorithms under different signal-to-noise ratios (SNR). Here, we used the reference algorithm for the least square (LS) algorithm, the orthogonal matching pursuit (OMP) algorithm, and the conventional SBL algorithm [22]. The user speed was 250 km/h, and $l_{\tau }=10$. As shown in Figure 5, in the case of a low signal-to-noise ratio, our proposed structured SBL algorithms have a significant advantage over other algorithms. Specifically, our algorithm better reduces interference.

**Figure 6:**
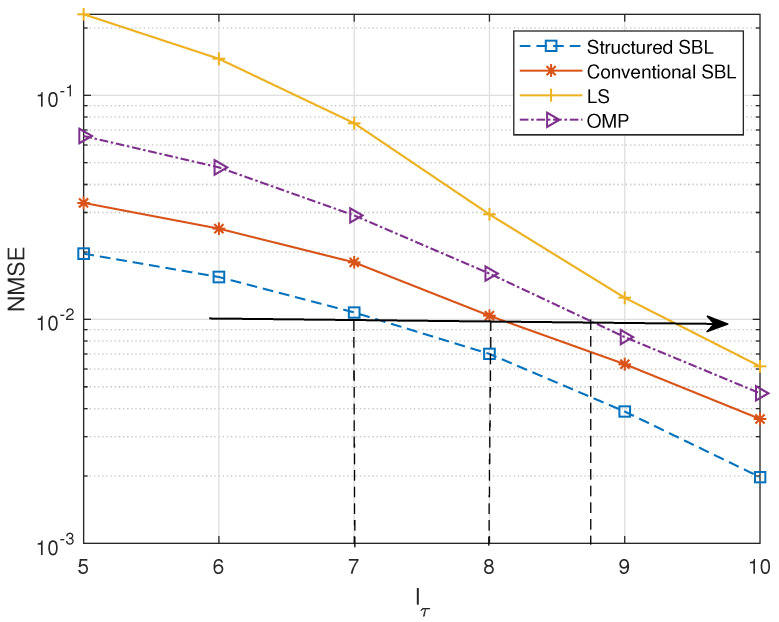
Delay range $l_{\tau }$ versus normalized MSE.

Then, we compared the cases where $l_{\tau }$ is constantly changing in different delay regions, as shown in Figure 6. In this OTFS modulation system, we set the SNR to 20 dB, the user speed to 250 km/h, and Q=4. The figure shows that the structured SBL algorithm we proposed maintains certain advantages. As the delay range $l_{\tau }$ continues to expand, the estimation results become increasingly accurate. We can see that under the same estimation error, compared to other algorithms, the structured SBL has the smallest value in delay dimension, which means that the pilot proportion is the smallest. For example, under the condition of a mean square error of $10^{-2}$, the values of the delay dimension $l_{\tau }$ of the structured SBL and conventional SBL algorithm are 7 and 8, respectively. The pilot overhead (except guard symbols) is $\eta =\frac{\left(2k_{\max}+1\right)\left(l_{\tau }+1\right)}{M\times N}$. The pilot overheads of the structured SBL and the conventional SBL are about 1.07% and 1.21%, respectively.

**Figure 7:**
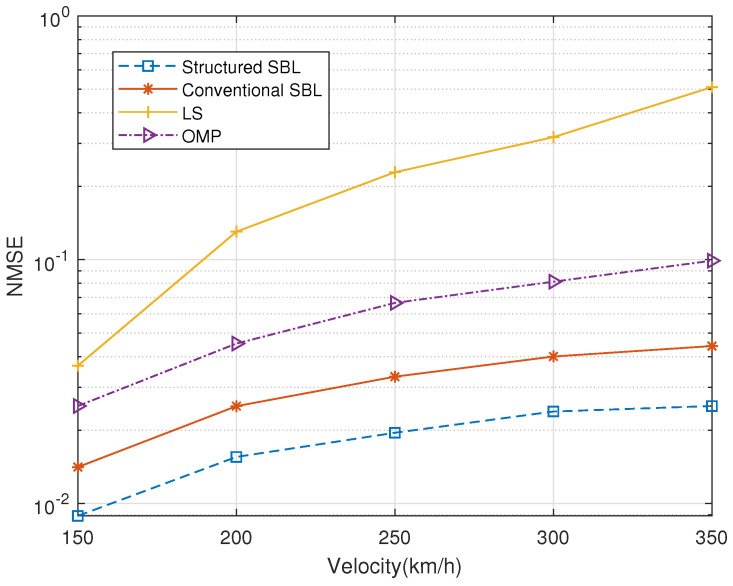
Velocity versus normalized MSE.

Next, we set the different speeds for performance comparison in Figure 7. Here, we set the speed variation range to [150, 350]; the SNR was 20 dB, Q=4, and $l_{\tau }=5$. The figure shows that the channel estimation results are affected to some extent with increasing speed. The higher the speed is, the slightly worse the accuracy of the estimation becomes. However, in terms of the trend of the curve, the curve is smooth overall with little change, and the LS algorithm is highly sensitive to changes in speed. On the other hand, the structured SBL algorithm maintains high robustness.

## 5. Conclusions

In this paper, we derived a sparse structure model formula from the input–output relationship of the DD domain. Based on the characteristics of the channel’s sparse structure, we treated channel estimation as a sparse recovery problem. In addition, we used block and continuous alternating optimization to solve the parameter problem of block distribution, which further improved the accuracy of channel estimation. Finally, through simulation analysis, we verified that the proposed algorithm has certain advantages over the traditional SBL algorithm. Under the condition of a low signal-to-noise ratio, the estimation accuracy was higher, and the influence of interference was diminished.

## Figures and Tables

**Table 1:** Simulation parameters.

Parameters	Value
Symbols *N*	64
Subcarries *M*	128
Carrier frequency Δf	15 kHz
Subcarrier frequency	$4\times 10^{9}$ Hz
The maximum delay $l_{max}$	10

## Data Availability

Not applicable.

## Appendix A

In an OTFS system, the input–output relation of DD domain is denoted as

(A1) $$\begin{array}{c} y\left[k,l\right]=\underset{Real\;channel}{\underset{︸}{\sum_{i=1}^{P}\sum_{q=-Q}^{Q}\left(\frac{e^{-j2\pi \left(-q-\kappa_{\nu_{i}}\right)}-1}{Ne^{-j\frac{2\pi }{N}\left(-q-\kappa_{\nu_{i}}\right)}-N}\right)h_{i}e^{-j2\pi \nu_{i}\tau_{i}}}}\times x\left[{\left[k-k_{\nu_{i}}+q\right]}_{N},{\left[l-l_{\tau_{i}}\right]}_{M}\right]+v\left[k,l\right] \end{array}$$

Another equation form is

(A2) $$y\left[k,l\right]=\sum_{k^{{}^{\prime }}=-k_{max}-Q}^{k_{max}+Q}\sum_{l^{{}^{\prime }}=0}^{l_{max}}h\left[k^{{}^{\prime }},l^{{}^{\prime }}\right]x\left[{\left[k-k^{{}^{\prime }}\right]}_{N},{\left[l-l^{{}^{\prime }}\right]}_{M}\right]$$

When $k^{{}^{\prime }}$ is fixed as constant, we have

(A3) $$y\left[k,l\right]=\sum_{l^{\prime }=0}^{l_{\max}}x_{k,l-l^{\prime }}^{T}h\left[l^{\prime }\right]$$

where

(A4) $$x_{k,l-l^{\prime }}^{}=\left[\begin{array}{c} x\left[k-\left(-k_{max}-Q\right),l-l^{\prime }\right]\\ \vdots \\ x\left[k-\left(k_{max}+Q\right),l-l^{\prime }\right] \end{array}\right]$$

(A5) $$h\left[l^{\prime }\right]=\left[\begin{array}{c} h\left[-k_{max}-Q,l^{\prime }\right]\\ \vdots \\ h\left[k_{max}+Q,l^{\prime }\right] \end{array}\right]$$

Finally,

(A6) $$y=\left[\begin{array}{c} y\left[k^{-},l^{-}\right]\\ \vdots \\ y\left[k^{+},l^{+}\right] \end{array}\right]=\left[\begin{array}{c} x_{k^{-},l^{-}}^{T}\\ \vdots \\ x_{k^{+},l^{+}}^{T} \end{array}\right]h=\Phi h+w$$

where $\left[k^{-},l^{-}\right]$ is the part of the estimation region.

## Appendix B

(A7)
$$\begin{array}{c} G\left(\alpha_{k},\gamma_{l},y|{\hat{\alpha }}_{k}^{\left(i\right)},{\hat{\gamma }}_{l}^{\left(i\right)}\right)=-\int p(h|y,{\hat{\alpha }}_{k}^{\left(i\right)},{\hat{\gamma }}_{l}^{\left(i\right)})\log\frac{p(\alpha_{k},\gamma_{l},h,y)}{p(h|y,{\hat{\alpha }}_{k}^{\left(i\right)},{\hat{\gamma }}_{l}^{\left(i\right)})}dh\\ ={}_{h|y,{\hat{\alpha }}_{k}^{\left(i\right)},{\hat{\gamma }}_{l}^{\left(i\right)}}\left[-\log\frac{p(\alpha_{k},\gamma_{l},h,y)}{p(h|y,{\hat{\alpha }}_{k}^{\left(i\right)},{\hat{\gamma }}_{l}^{\left(i\right)})}\right]\\ \geq -\log p(\alpha_{k},\gamma_{l},y)\\ =-\log\left(\int p(h|y,{\hat{\alpha }}_{k}^{\left(i\right)},{\hat{\gamma }}_{l}^{\left(i\right)})\log\frac{p(\alpha_{k},\gamma_{l},h,y)}{p(h|y,{\hat{\alpha }}_{k}^{\left(i\right)},{\hat{\gamma }}_{l}^{\left(i\right)})}\right)\\ =-\log p(\alpha_{k},\gamma_{l},y) \end{array}$$
